# Computational Study of Hemodynamic Field of an Occluded Artery Model with Anastomosis

**DOI:** 10.3390/bioengineering10020146

**Published:** 2023-01-21

**Authors:** Panagiotis Parissis, Alexandros Romeos, Athanasios Giannadakis, Alexandros Kalarakis, Michail Peroulis

**Affiliations:** 1 Department of Mechanical Engineering and Aeronautics, Laboratory of Applied Thermodynamics, University of Patras, 26504 Patras, Greece; 2 Department of Mechanical Engineering, Laboratory of Heating Cooling and Refrigeration, University of the Peloponnese, 26334 Patras, Greece; 3 Department of Mechanical Engineering, Laboratory for Analysis of Materials and Structures, University of the Peloponnese, 26334 Patras, Greece; 4 Department of Surgery—Vascular Surgery Unit, Faculty of Medicine, University of Ioannina, 45110 Ioannina, Greece

**Keywords:** stenosis, anastomosis, hemodynamics, turbulence flow models, computational fluid dynamics, experimental visualizations

## Abstract

In this research work, the hemodynamic field of an occluded artery with anastomosis by means of computational simulation has been studied. The main objective of the current study is the investigation of 3D flow field phenomena in the by-pass region and the effect of the bypass graft to stenosis volume flow ratio on their formation. The anastomosis type was end-to-side with a 45° angle, while stenosis imposed a 75% area blockage of the aorta vessel and the total volume flow was 220 lt/h. The computational study of the flow field was utilized via a laminar flow model and three turbulence models (k—ε RNG, standard k—ω, and k—ω SST). Numerical results were compared qualitatively with experimental visualizations carried out under four different flow conditions, varying according to the flow ratio between the stenosis and the anastomotic graft. Comparison between computational results and experimental visualization findings exhibited a good agreement. Results showed that SST k—ω turbulence models reproduce better visually obtained flow patterns. Furthermore, cross-sectional velocity distributions demonstrated two distinct flow patterns down the bypass graft, depending on the flow ratio. Low values of flow ratio are characterized by fluid rolling up, whereas for high values fluid volume twisting was observed. Finally, areas with low wall shear stresses were mapped, as these are more prone to postoperative degradation of the bypass graft due to the development of subendothelial hyperplasia.

## 1. Introduction

A large number of deaths are recorded worldwide annually due to cardiovascular diseases. Nearly 31% of all deaths are related to atherosclerosis, a disease responsible for unusual hemodynamic conditions in the arteries [1]. Atherosclerosis is a silent, multifactorial and complex disease that is defined as the accumulation of lipids and immune cells in the arterial wall, resulting in the formation of a plaque [2,3]. This phenomenon has been correlated to local biomechanical and biological factors [4,5,6], with the leading cause being local hemodynamics [7,8,9,10,11,12]. A well-defined predictor of coronary atherosclerosis progression is wall shear stress (WSS). However, despite the fact that low WSS regions have been commonly recognized as prone to plaque development, high values of WSS are related to plaque destabilization [13,14,15,16,17]. This pathology causes serious damage when it develops in coronary arteries, because, the agglomeration of fatty materials creates an occlusion, which affects the normal blood flow to the heart, leading to a limited supply of blood, oxygen, and other vital nutrients fundamental for its normal function. Gradually, the restricted blood flow could cause chest pain or a heart attack when the artery gets completely blocked [18,19].

In the case of a narrowing artery, several surgical techniques have been developed to address this vessel‘s stenosis. Furthermore, new evidence has been brought to light due to a recent study which proved that patients with COVID-19 are at high risk for thrombotic arterial and venous occlusions. The atherosclerosis development in the background of the increased mortality rate caused by the virus reveals a complex situation that requires further investigation [20]. This pathology is usually treated with balloon angioplasty and placement of an intracoronary stent, nevertheless, there is a high probability of artery restenosis [21,22]. For cases where the stenosis exhibits high obstruction (>75%) of the lumen of the artery, bypassing the stenosis is the preferred treatment method. The anastomosis of the affected vessel with other healthy vascular or artificial implants restores its perfusion and eventually the smooth functioning of the heart’s circulatory system.

However, a significantly high failure rate of specific occlusion vessel bypass procedures associated with the longevity of anastomosis implants is observed postoperatively. The main causes for the occurrence of diseases in the area of graft-sealed vessel sutures are surgical injuries [23], the incompatibility of the materials [24], and the abnormal configuration of the hemodynamic field in the area of the anastomosis [25]. Among the major problems that have been addressed in the postoperative progression of the grafts are the occurrence of subendothelial hyperplasia or thrombosis in the area of anastomosis, diseases that cause long-term vascular narrowing, dysfunction, and eventual failure.

The widespread use of the obstruction bypass method has increased the research interest to extend the life expectancy of the vascular graft and elucidate the parameters that play a key role in a successful anastomosis process. Focusing on the study of the hemodynamic field, several parameters arise, mainly related to the effect of the anastomosis geometry, and appear to play an important role in long-term grafting. The forces developed in the hemodynamic field promote the reshaping of the blood vessels [26]. Several studies have examined the effect of forces exerted on the vessel walls on the occurrence of subendothelial hyperplasia in vascular implants [23,24,27,28,29,30,31]. The results of these studies demonstrated that the effects of wall shear stress contribute to the occurrence of post-endothelial hyperplasia. It has been shown that the arteries tend to adjust their geometry in order to maintain consistent shear stress levels on their walls [32].

In the case of end-to-side anastomosis, the correlation of the occurrence of subendothelial hyperplasia with shear stress patterns developed in the artery walls remain not fully understood. This results from the complex hemodynamic field in the area of anastomosis, which is characterized by the formation of turbulent structures due to separation, recirculation zones as well as stationary regions. The interaction of these flow patterns is related to the development of subendothelial hyperplasia [33,34,35]. Low shear stress levels accompanied by steep gradients of their distributions in the anastomosis area are the characteristic indicators for the diagnosis of the occlusion of the vascular graft. On the other hand, the presence of unexpectedly high shear stress values has been associated with premature thrombosis and graft failure, which is largely related to the sub dimensioning of the vascular graft [36]. The key parameters affecting the hemodynamic field in the area of anastomosis are the intrusion angle and the level of admission of the graft [28,37,38,39], the relative positioning of the anastomosis with respect to the artery stenosis [40] and the graft/artery diameter ratio [39]. The choice of a specific anastomosis configuration is a complex procedure due to the effect of each of the aforementioned factors on the local hemodynamic field but also on the surgical feasibility of each proposed solution.

Taking into consideration the global impact of this disease, it is of great importance to acquire deeper knowledge about blood flow hemodynamics [1], which is the main aim of this research work. In the current manuscript, we studied the hemodynamic flow field in a by-pass region of an occluded artery model is being studied with experimental visualizations and numerical simulations. Important results were extracted for the rolling up and separation flow. Moreover, the flow ratio from stenosis/anastomosis regions and the regions that presented high and low values of wall shear stresses in the arterial walls were also determined. Finally, the comparison of experimental and numerical results about the turbulence flow model that provides better results was further elucidated

## 2. Materials and Methods

### 2.1. Model Geometry

**Figure 1:**
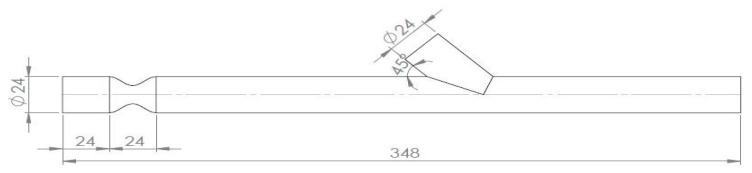
Geometry sketch.

The aim of this study was the comparison the hemodynamic field in the by-pass region from numerical and experimental results. In order to accomplish that a geometry based on the experimental domain was created. A sketch of the geometry is presented in Figure 1. 

**Figure 2:**
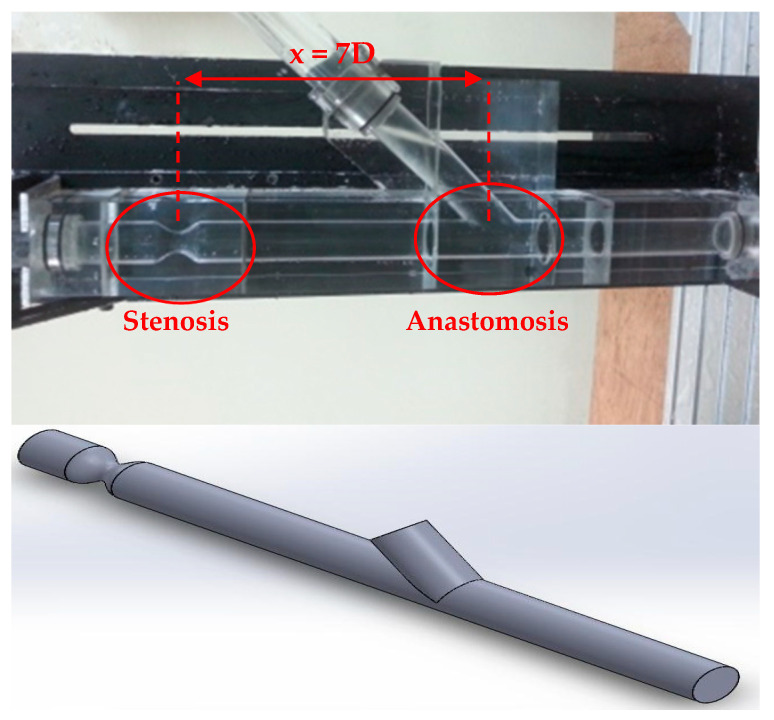
Experimental part and 3D geometry of the aorta with stenosis and anastomosis regions.

The artery model had an internal diameter of D = 24 mm and a 75% area blockage ratio, leading to an internal stenosis diameter of 12 mm, while the anastomosis angle was 45°. The anastomosis region was 7D and the whole vessel 12.5D ahead from the middle of stenosis, as it is demonstrated in Figure 2.

As already mentioned before the 3D geometry was based on the experimental setup and created with the Dassault Systems SolidWorks. Figure 2 demonstrates the experimental part of the aorta with stenosis and anastomosis regions and the 3D geometry that was generated.

The stenosis curves are very important design parameters that have to be mentioned. The artificial geometry model of the stenosis region is axisymmetric, and both experimental and computational geometry have been created using the equations below (Equations (1)–(3)).

(1) $$F\left(x\right)=x_{0}\pm 3\cdot {\left[\cos\left(\frac{\pi \cdot x}{12}\right)\right]}_{\;}$$

where x_0_ is the point for placing the respective geometric equation which depends on the position and geometry of the stenosis, x is the length point for the calculation of y coordinate point.

**Figure 3:**
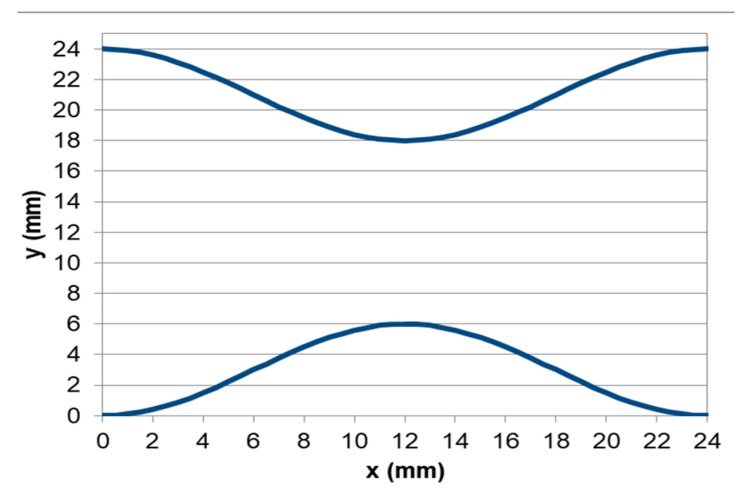
The two curves of stenosis.

For this study, the x_0_ term had to be equal to 21 and 3 respectively for the creation of the two curves, so that F_1_(0) = 24 mm and F_2_(0) = 0 mm. The stenosis curves are presented in Figure 3.

(2) $$F_{1}\left(x\right)=21+3\cdot \left[\cos\left(\frac{\pi \cdot x}{12}\right)\right]$$

(3) $$F_{2}\left(x\right)=3-3\cdot \left[\cos\left(\frac{\pi \cdot x}{12}\right)\right]$$

### 2.2. Governing Equations

Three-dimensional Navier–Stokes conservation of mass and momentum equations for incompressible flow were solved (Equations (4) and (5)) in order to calculate the flow field in the computational domain.

(4) $$\nabla (\rho \vec{u})=0$$

(5) $$\frac{\partial }{\partial t}(\rho \vec{u})+\nabla (\rho \vec{u}\vec{u})=-\nabla p+\nabla \left[\mu \left[\left(\nabla \vec{u}+\nabla {\vec{u}}^{T}\right)-\frac{2}{3}\nabla \vec{u}I\right]\right]$$

where *I,* is the unit tensor, u, p, μ, and ρ are the flow velocity, flow pressure, blood dynamic viscosity, and density, respectively.

In this study, we tested three Reynolds Averaged Navier-Stokes (RANS) turbulence models. The k—ε RNG, the standard k—ω, and the k—ω SST.

The k—ε RNG turbulence model has two transport equations for the turbulence kinetic energy k and its rate of dissipation ε as described by equations 6 and 7.

(6) $$\frac{\partial }{\partial }\left(\rho k\right)+\frac{\partial }{\partial x_{i}}\left(\rho ku_{i}\right)=\frac{\partial }{\partial x_{j}}\left(\alpha_{k}\mu_{eff}\frac{\partial k}{\partial x_{j}}\right)+G_{k}+G_{b}-\rho \varepsilon -Y_{M}$$

(7) $$\frac{\partial }{\partial }\left(\rho \varepsilon \right)+\frac{\partial }{\partial x_{i}}\left(\rho \varepsilon u_{i}\right)=\frac{\partial }{\partial x_{j}}\left(\alpha_{\varepsilon }\mu_{eff}\frac{\partial \varepsilon }{\partial x_{j}}\right)+C_{1\varepsilon }\frac{\varepsilon }{k}\left(G_{k}+C_{3\varepsilon }G_{b}\right)-C_{2\varepsilon }\rho \frac{\varepsilon^{2}}{k}$$

where $G_{k}$ represents the generation of turbulence kinetic energy due to the mean velocity gradients. $G_{b}$ is the generation of turbulence kinetic energy due to buoyancy. $Y_{M}$ represents the contribution of the fluctuating dilatation in compressible turbulence to the overall dissipation rate. The quantities $\alpha_{k}$ and $\alpha_{\varepsilon }$ are the inverse effective Prandtl numbers for k and ε, respectively, C_1ε_ = 1.42 and C_2ε_ = 1.68.

Equation (8) integrated to obtain an accurate description of how the effective turbulent transport varies with the effective Reynolds number (or eddy scale), allowing the model to better handle low-Reynolds number and near-wall flows.

(8) $$d\left(\frac{\rho^{2}k}{\sqrt{\varepsilon \mu }}\right)=1.72\frac{\hat{\nu }}{\sqrt{{\hat{\nu }}^{3}-1+C_{\nu }}}d\hat{\nu }$$

where $\hat{\nu }$ = μ_eff_/μ and $C_{\nu }$ ≈ 100.

The standard k—ω turbulence model of Wilcox has two transport equations for the turbulence kinetic energy k and the specific dissipation rate as follows (Equations (9) and (10)).

(9) $$\frac{\partial }{\partial }\left(\rho k\right)+\frac{\partial }{\partial x_{i}}\left(\rho ku_{i}\right)=\frac{\partial }{\partial x_{j}}\left(\Gamma_{k}\frac{\partial k}{\partial x_{j}}\right)+G_{k}+G_{b}-Y_{\kappa }$$

(10) $$\frac{\partial }{\partial }\left(\rho \omega \right)+\frac{\partial }{\partial x_{i}}\left(\rho \omega u_{i}\right)=\frac{\partial }{\partial x_{j}}\left(\Gamma_{\omega }\frac{\partial \omega }{\partial x_{j}}\right)+G_{\omega }+G_{\omega b}-Y_{\omega }$$

where $G_{k}$ represents the generation of turbulence kinetic energy due to mean velocity gradients. $G_{\omega }$ represents the generation of ω. $\Gamma_{k}$ and $\Gamma_{\omega }$ represent the effective diffusivity of k and ω, respectively. $Y_{\kappa }$ and $Y_{\omega }$ represent the dissipation of k and ω due to turbulence.

For low-Reynolds number corrections, the coefficient damps the turbulent viscosity causing a low-Reynolds number correction and it is given by Equation (11).

(11) $$\alpha^{*}=\alpha_{\infty }^{*}\left(\frac{\alpha_{0}^{*}+\frac{Re_{t}}{R_{k}}}{1+\frac{Re_{t}}{R_{k}}}\right)$$

where Re_t_ = ρk/(μω), R_k_ = 6, $\alpha_{0}^{*}$ = β_i_/3 and β_i_ = 0.072.

The shear stress transport (SST) k—ω turbulence model was developed by Menter to blend the robust and accurate formulation of the standard k—ω model in the near-wall region with the freestream independence of the k—ε model in the far field. To achieve this, a proper transport behavior by a limiter to the formulation of the eddy-viscosity was inserted [41].

### 2.3. Numerical Model

**Figure 4:**
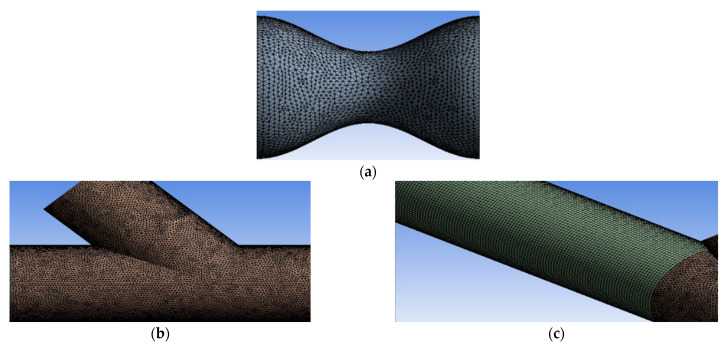
(**a**) and (**b**) grid sections with tetrahedral elements, (**c**) grid segment with hexahedral elements.

In the current study, numerical models by the commercial software ANSYS Fluent have been used. Figure 4 depicts the hybrid mesh that was created in ANSYS Meshing, which contains hexahedral elements in the artery region and tetrahedral elements in stenosis and anastomosis regions. Near the walls, the refinement regions ensured an appropriate computational grid number of elements and achieved calculation accuracy.

### 2.4. Grid Sensitivity Analysis

In order to ensure the accuracy of the results, four different grids were created and tested. The grids and the number of elements that have been used are presented in Table 1. The simulations used for comparing the different meshes were made for steady-state flow conditions with Q = 220 lt/h, a flow rate ratio of 47%/53% between the stenosis/anastomosis streams and the k—ε RNG turbulence flow model was selected for these simulations.

**Figure 5:**
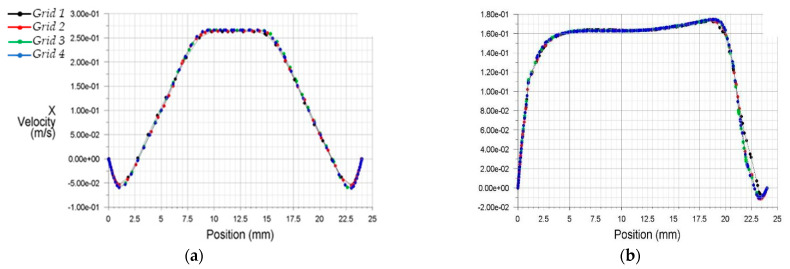
Streamwise x velocity profiles at two downstream stations from the stenosis (**a**) 0.5D, (**b**) 8.0D.

Characteristic results obtained from the different grid configurations at the central vertical plane of the flow are shown in Figure 5. This figure depicts distributions of the longitudinal x velocity at two different downstream distances from the center of the stenosis (0.5D and 8D).

**Figure 6:**
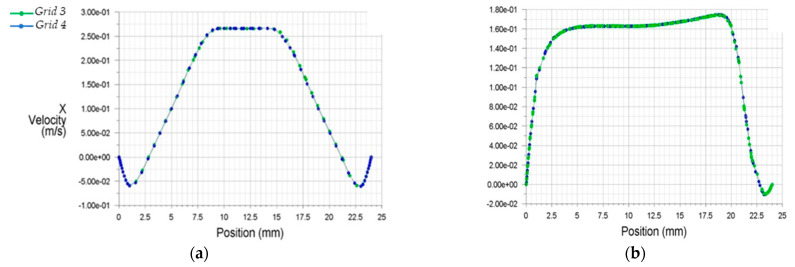
Streamwise x velocity profiles for two condensed grids at two downstream stations from the stenosis (**a**) 0.5D, (**b**) 8.0D.

The results of discretization 3 and 4 (Grids 3 and 4) appeared more appropriate to analyze the internal flow dynamics than the former two grid selections (Grids 1 and 2). For better optimization, Figure 6 demonstrates the x velocities profiles from Grids 3 and 4.

Figure 6 clarifies the accuracy of the results for both grids selections. Due to the above, for the final computational investigation, Grid 3, which consisted of 2.453.817 elements and 1.228.433 nodes, was selected, as it led to faster convergence and required less computational power than Grid 4.

### 2.5. Boundary Conditions

To an original cardiovascular system, the flow is pulsatile, although it is quite common to study steady-state relevant flow as many experimental and computational works have been performed in order to have a perception of time-dependent flow dynamics. The steady-state situation is quite similar to the systolic phase of the pulsatile flow [34].

The SIMPLE algorithm was used for pressure-velocity coupling of the governing equations. The pressure, momentum, and turbulence equations are spatially discretized using the second-order upwind scheme.

In large arteries, such as aorta, blood can be assumed as a Newtonian fluid [26]. Therefore, blood has been studied as incompressible and Newtonian fluid. For the experimental approach, blood was simulated via a mixture of water-glycerin (55–45%) with a dynamic viscosity of μ = 3.35 cP similar to that of blood (μ_blood_ = 3.5 cP) and density of ρ = 1060 kg/m^3^. Numerical simulations were conducted assuming the same blood properties, with steady flow inlet conditions, with total flow rate of Q = 220 lt/h.

**Figure 7:**
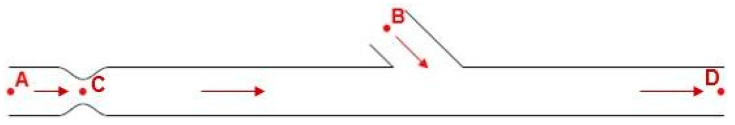
Area points of calculations.

Table 2 demonstrates the flow rate ratio scenarios that have been tested with the calculations of local velocity magnitude and Reynolds number for characteristic domain cross sections as demonstrated in Figure 7. As seen in Figure 7 the simulation model had two inlets. The first was located upwards of the stenosis (position A) and the second one was located in the bypass graft (position B). Fixed velocity boundary condition has been used for both inlets while fixed pressure boundary condition (zero-gauge pressure) has been used for the model’s outlet (position D).

## 3. Results

### 3.1. Qualitative Comparison of Flow Models

**Figure 8:**
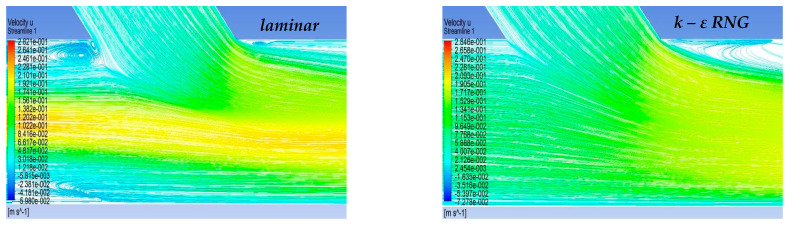

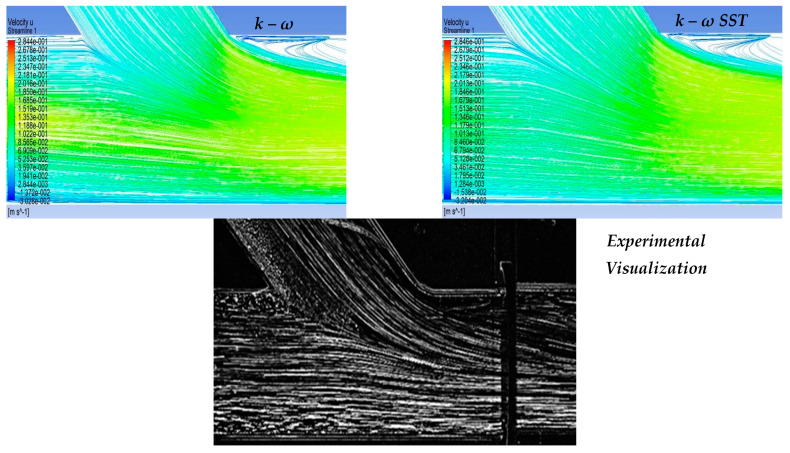
Qualitative comparison of flow models for 47–53% stenosis/anastomosis flow rate ratio.

**Figure 9:**
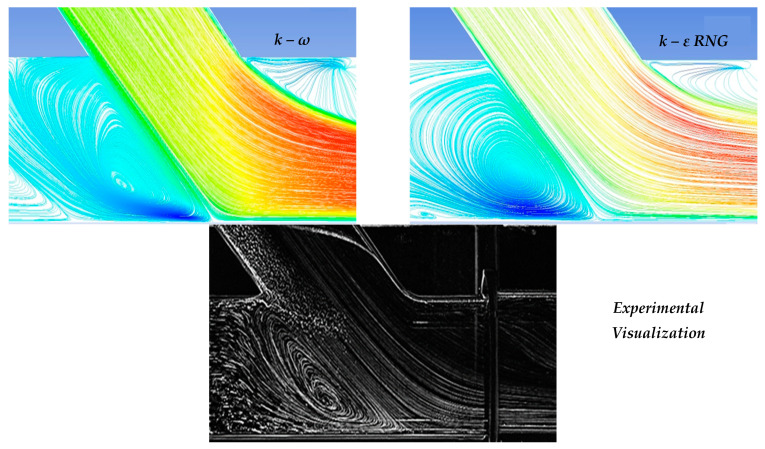
Qualitative comparison of flow models for 0–100% stenosis/anastomosis flow rate ratio.

In the context of the present study, four cases of stenosis to anastomosis flow rate ratio were studied (Table 2). In Figure 8 and Figure 9, streamwise velocity contours, in conjunction with flow field streamlines for all flow models (laminar, k-ε RNG, k-ω and k-ω SST), are presented. The results shown in Figure 3 and Figure 4 account for the steady state flow conditions with a total flow rate of 220 lt/h and stenosis/anastomosis stream ratios of 47% -53% and 0% -100%, respectively. The simulated flow regimes are then compared with the corresponding experimental visualization data [34,35].

A comparison of computational simulations with visualization revealed that the k—ε RNG model did not respond sufficiently to any of the four cases under investigation. A typical example was the 0–100% stenosis/anastomosis case, where the recirculation zone upstream of the impinging by-pass jet had no qualitative agreement with the experimental visualization. Regarding the laminar model output, it was concluded that the computational results are acceptable on a case-by-case basis. For the 47–53% ratio, the model did not respond satisfactorily, while primary/secondary flow ratios results were acceptable.

Results from the standard k—ω flow model simulation appeared to show the best correlation with the experimental visualization when compared with other models for all studied cases. The choice of this model has been also supported by related literature [42,43,44,45,46,47,48,49,50,51] as a reliable selection for resolving flow fields exhibiting intense recirculation phenomena.

Finally, the k-ω SST flow model computational results were acceptable and qualitatively identical to the conducted experimental visualizations. In addition, the computational results of this flow model were indistinguishable from the corresponding results obtained by the k—ω model. Therefore, both k-ω turbulent flow models are considered reliable and suitable for simulating the merging flow configurations considered in this study. However, a further study aiming at the quantitative comparison of the experimental and numerical results is required in order to clarify which of these two k—ω turbulent models works out best.

### 3.2. Velocity Field in the Artery Anastomosis Region

**Figure 10:**
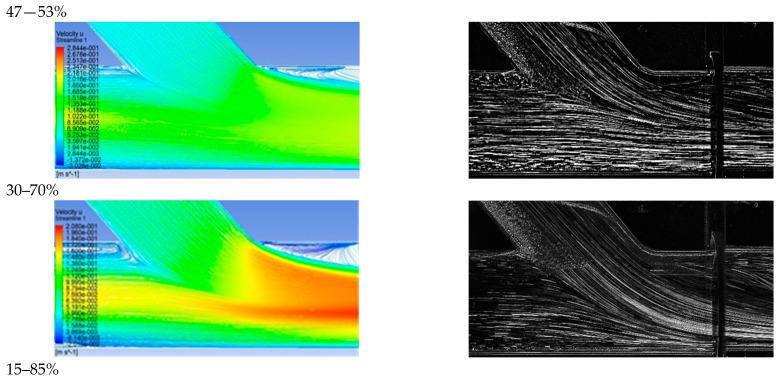

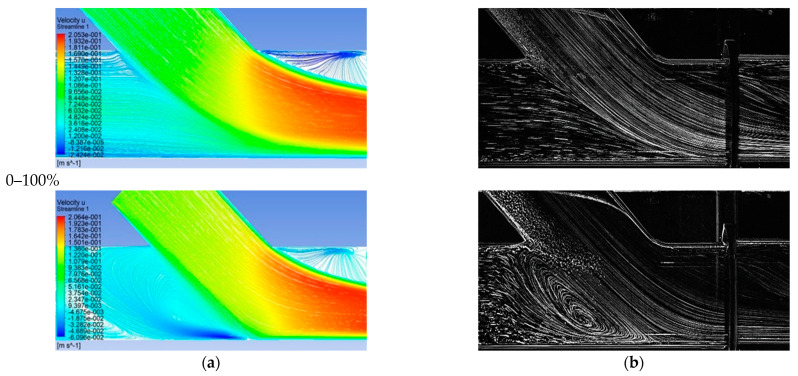
Comparison between computational and experimental visualization results in the anastomosis region (**a**) Computational results of the longitudinal velocity component, (**b**) experimental visualization results.

Figure 10 depicts the comparison between computational and visualization experiment results for the four stenosis/anastomosis flow ratio scenarios that have been tested (Ratios 47–53%, 30–70%, 15–85%, 0–100%). A detailed description of these cases is given in Table 2.

Figure 10a shows the results of the longitudinal velocity component obtained from the standard k—ω turbulence model. In all cases, a recirculation area was observed at the anastomosis heel due to the blockage of the duct upper boundary layer produced by the anastomosis graft flow. Flow separation and rolling up flow occurred on the opposite side of the bypass graft, at the toe region. The axial velocities were higher in the lower half of the main tube in a similar manner to that found when a developed flow enters a curved bend.

Velocity maxima accompanied by steep radial velocity gradients and increased shear stresses took place in the near bed zone of the artery and along its distal direction. Flow separation and flow rolling up were observed at the toe, which is an undesirable effect as it can lead to material deposition (lipids etc) and potential graft failure in this region. Flow slowdown could be detected after the toe tip in the upper host vessel zone, with clear stagnation zones depicted 1D downwards the anastomosis.

The absence of flow from stenosis ($\dot{m}$_by-pass_ = 100%) led to the impingement of fluid on artery walls as it inserted the aorta via the bypass graft, while the existence of stagnant fluid located downwards the stenosis resulted in the creation of a wide recirculation zone, accompanied by correspondingly high levels of vorticity and shear of the flow field. Additionally, high values of mean longitudinal and radial velocities were observed in the impingement and infusion regions of the flui, respectivelyy.

The recirculation zone vanished for low flow operation of the bypass graft. In the case of flow ratio, $\dot{m}$_by-pass_ = 85% intense flow mixing was observed but with lower levels of vorticity and shear when compared to the previous case, still strong fluid impingement appeared on artery walls as it emerged from the bypass graft.

In the case of the flow ratio of $\dot{m}$_by-pass_ = 70% the topology of the flow field showed significant change. In this scenario, there was no fluid impingement on artery walls because of the high amount of flow emerging from the stenosis region, occupying 25% of the artery cross-section area in the mixing region of the two flows. In this region, high levels of shear were observed due to the two flow interactions and subsequent acceleration of the fluid from the stenosis region, due to the reduction of the effective area in which the fluid flows.

Finally, in the case of flow ratio $\dot{m}$_by-pass_ = 53%, the topology of the flow field is almost the same as in the previous case with the flow ratio of $\dot{m}$_by-pass_ = 70% but with smaller levels of shear due to a higher ratio of the flow from the stenosis region that leads to a 50% occupation of the artery area in the mixing region of two flows.

### 3.3. Cross Sectional Velocity Distribution

**Figure 11:**
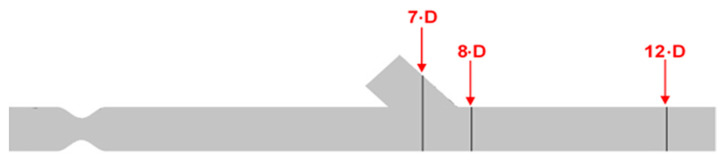
Identification of the cross sections for computational result presentation along with the artery model.

Figure 11 depicts the positions of the previously mentioned cross sections.

**Figure 12:**
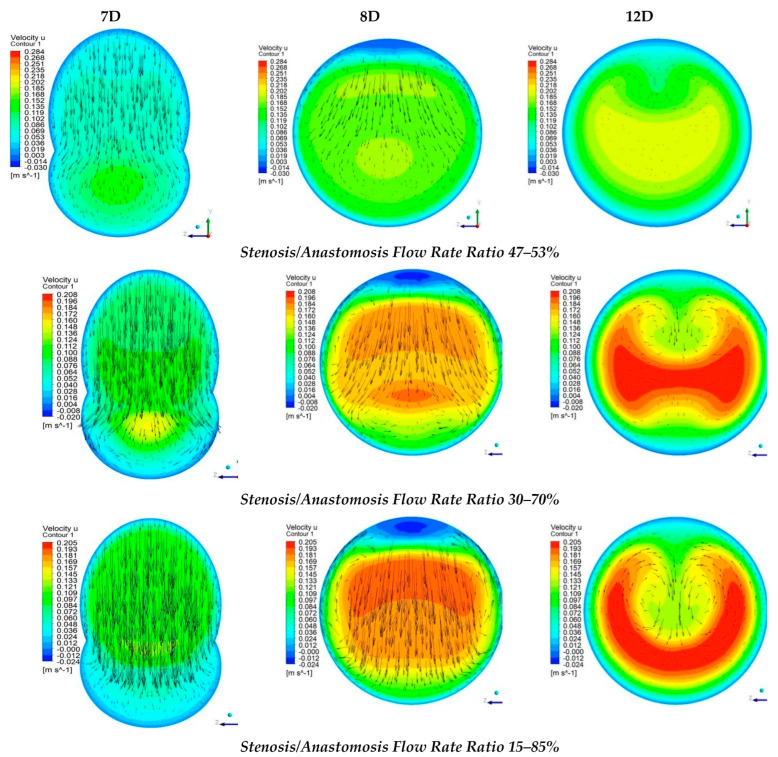

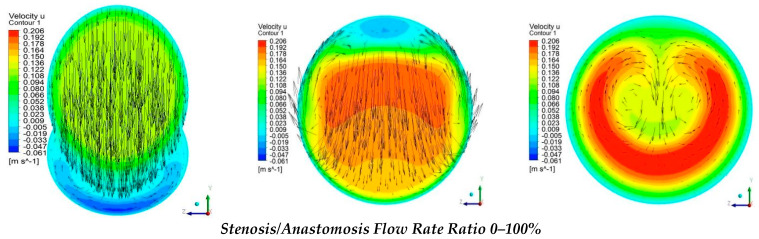
Streamwise velocity distribution in three cross sections with different stenosis/anastomosis flow rate ratios.

Figure 12 shows numerical results of the longitudinal velocity distributions at various transverse sections downwards the anastomosis (7D, 8D, and 12D), for all cases of stenosis/anastomosis flow ratios which have been studied.

The results from Figure 12 elaborated that in these four cases, there were two different flow regimes. For the cases where $\dot{m}$_by-pass_ = 100% and 85%, the flow formed a rolling up due to the fact that the flow field in both cases was formed mainly by the bypass graft. Moreover, as expected, in the case of $\dot{m}$_by-pass_ = 100%, the flow rolling up was more intense than that of $\dot{m}$_by-pass_ = 85%.

As already mentioned, in cases with flow ratios of $\dot{m}$_by-pass_ = 70% and 53% the topology of the flow field was quite different. In these two cases, the flow from stenosis occupied a significant amount of the aorta area and down the anastomosis region, the flow twisted. More specifically, when the flow ratios of $\dot{m}$_by-pass_ = 70% the topology change of the flow field in contrast with the two previous cases was related to the fact that twisting characteristics of the flow appear in the mixing area of the two flows. with respect to the axis of symmetry of the vessel, in which the transverse component of velocity takes the same measure but opposite sign. In the case of $\dot{m}$_by-pass_ = 53%, flow twisting became more pronounced.

### 3.4. Wall Shear Stresses (WSS)

**Figure 13:**
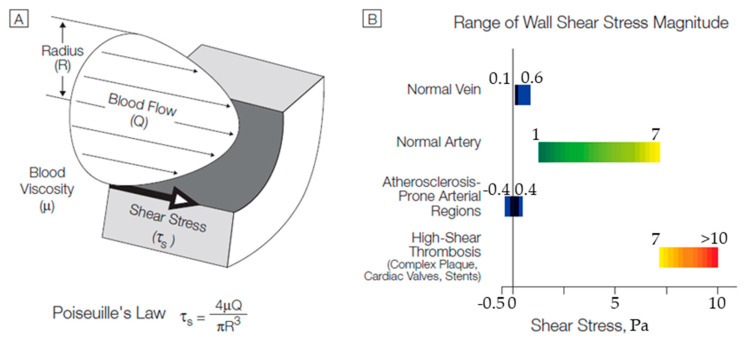
(**A**) Cross-sectional schematic diagram of a blood vessel illustrating hemodynamic shear stress, τ_s_, the frictional force per unit area acting on the inner vessel wall and on the luminal surface of the endothelium as a result of the flow of viscous blood. (**B**) Tabular diagram illustrating the range of shear stress magnitudes encountered in veins, arteries, and in low-shear and high-shear pathologic states. Adapted from Malek [52].

Figure 13 demonstrates a tabular diagram that illustrates the range of shear stress magnitudes encountered in veins, arteries, and in low-shear and high-shear pathologic states.

**Figure 14:**
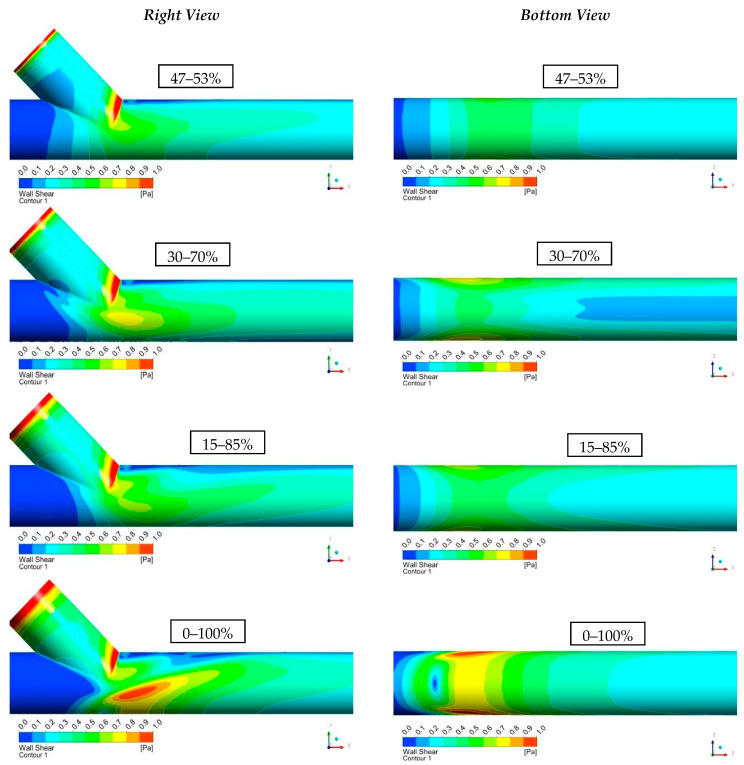
Results of Shear Stresses on the Vessel Walls (WSS).

Figure 14 shows the computational results of the shear stresses on the vessel walls (WSS). It is observed that, in all cases, the levels of shear stresses were low (WSS < 1 Pa). It is worth mentioning that in all scenarios, the highest values of WSS were located at regions where intense recirculation takes place or in conjunction with the vessels. Finally, as blood flow from the vascular graft increased compared to that of the occluded artery, it resulted in the elevation of the shear stress levels at the bottom of the artery.

## 4. Conclusions

The qualitative comparison of numerical results derived from k—ω turbulence models, with experimental visualizations, exhibited a significant agreement. However, a quantitative comparison must be performed in order to declare which k—ω model (standard or SST) is the most appropriate for this study.

The current study focused on the velocity flow field in the anastomosis region and indicated separation, recirculation, and rolling up zones, depending on which flow scenario was investigated. Additionally, velocity distribution in cross-sections demonstrated two different flow regimes. Depending on the mass flow ratio, the flow down the anastomosis rolled up or twisted when the flow from the stenosis region was low or high, respectively. In the same manner, regions with low wall shear stress analysis were depicted, thereby illuminating areas that were more prone to develop subendothelial hyperplasia.

In conclusion, this research study provides a deeper understanding of the flow phenomena developed in the anastomosis region. Still, further experimental/numerical study is needed to be performed, taking into account the elasticity and 3D geometry of arteries and bypass grafts.

## Figures and Tables

**Table 1:** Number of Elements and Nodes for the Tested Numerical Grids.

Grids	Number of Elements	Number of Nodes
Grid 1	7.65·10^5^	4.28·10^5^
Grid 2	1.15·10^6^	5.81·10^5^
Grid 3	2.45·10^6^	1.23·10^6^
Grid 4	3.17·10^6^	1.63·10^6^

**Table 2:** Test case scenarios with calculation of velocities and Reynolds numbers.

Cases	Flow Rate Proportion	Calculation Positions	Velocity (m/s)	Re
1st	47%	A	0.063	482
	53%	B	0.072	544
	47%	C	0.254	964
	100%	D	0.135	1026
2nd	30%	A	0.041	308
	70%	B	0.095	718
	30%	C	0.162	616
	100%	D	0.135	1026
3rd	15%	A	0.02	154
	85%	B	0.115	872
	15%	C	0.081	308
	100%	D	0.135	1026
4th	0%	A	0	0
	100%	B	0.135	1026
	0%	C	0	0
	100%	D	0.135	1026

## Data Availability

The research data will be available after contact with the authors.
